# Comparison of Vie Scope and Macintosh Laryngoscope in Adults With an Expected Easy Airway: A Randomized Controlled Trial

**DOI:** 10.7759/cureus.88566

**Published:** 2025-07-23

**Authors:** Vasileios I Boviatsis, Alexios Triantopoulos, Abraham Pouliakis, Dimitra Boviatsi, Theodoros Xanthos, Nicoletta Iacovidou, Konstantinos Ekmektzoglou

**Affiliations:** 1 Department of Anesthesiology, General Hospital of Patras, Patras, GRC; 2 Department of Pathology, National and Kapodistrian University of Athens, Athens, GRC; 3 Department of Radiology, Children's Hospital P. and A. Kyriakou, Athens, GRC; 4 Department of Medicine, European University Cyprus, School of Medicine, Nicosia, CYP; 5 Department of Neonatology, National and Kapodistrian University of Athens, Athens, GRC; 6 Department of Gastroenterology, Army Share Fund Hospital (NIMTS), Athens, GRC

**Keywords:** airway management, cardiopulmonary resuscitation, endotracheal intubation, laryngoscopes, respiratory system, vocal cords

## Abstract

Background: The technique of visualizing the vocal cords during direct laryngoscopy has made the Macintosh laryngoscope (HEINE Optotechnik GmbH & Co. KG, Gilching, Germany) the optimal choice for endotracheal intubation since the middle of the 20th century. On the other hand, the use of full personal protective equipment during the SARS-CoV-2 pandemic impeded the successful completion of medical procedures, such as endotracheal intubation during cardiopulmonary resuscitation in patients infected with COVID-19. These circumstances necessitated the development of a new type of laryngoscope, the Vie Scope (Adroit Surgical LLC, Oklahoma City, OK, USA).

Materials and methods: A single-blind, randomized, prospective, superiority clinical trial was conducted. Patients with an expected easy airway were enrolled and allocated in a 1:1 ratio to either the Vie Scope or Macintosh. The first-attempt intubation success rate and the required intubation time were defined as primary outcome measures, while the overall intubation success rate and glottis visualization, as classified by the Cormack-Lehane scale, were considered secondary outcomes.

Results: This study included 264 patients. The required intubation time was shorter with the Macintosh than with the Vie Scope (median (interquartile range (IQR)): 7.80 (1.50) and mean (standard deviation (SD)): 8.08 (5.34) seconds vs. median (IQR): 15.65 (3.90) and mean (SD): 16.65 (5.02) seconds, difference in means: 8.60 seconds, 95% CI: -∞ to 7.9, p<0.0001). The first-attempt intubation success rate was estimated at 100% (132/132) and 90.15% (119/132) using the Macintosh and Vie Scope, respectively (relative risk: 0.90 (=1/1.1, 95% CI: 0.85-0.95), p=0.0002). Furthermore, a higher overall intubation success rate was noted with the Macintosh (132/132, 100%) than with the Vie Scope (124/132, 93.94%), p=0.0041. No statistically significant difference was detected between the laryngoscopes regarding the degree of the glottis visualization (p=0.7895).

Conclusions: The first-attempt and overall intubation success rates, as well as the required intubation time with the Macintosh, were superior to those with the Vie Scope when used for the establishment of anticipated easy airways, without a statistically significant difference in glottis visualization grade.

## Introduction

Preserving the airway’s patency through endotracheal intubation is crucial for both elective general anesthesia and emergency airway management [1,2]. Given the indirect elevation of the epiglottis by a curved blade, the Macintosh laryngoscope (HEINE Optotechnik GmbH & Co. KG, Gilching, Germany) remains a preferred and reliable choice for endotracheal intubation, despite the widespread use of a videolaryngoscope, especially in difficult airway settings [3,4]. On the other hand, the illuminated, enclosed, straight tube with an angled end of the Vie Scope (Adroit Surgical LLC, Oklahoma City, OK, USA) enables endotracheal intubation via the VOIR Bougie (Adroit Surgical LLC, Oklahoma City, USA), warranting its inclusion as a method for managing difficult airways in both inpatient and outpatient settings [5,6]. In particular, the Vie Scope is characterized by a comparable or even higher mean intubation time, a successful intubation rate, and glottis visualization comparable to other laryngoscopes, such as the Macintosh and other videolaryngoscopes [7,8].

However, the current literature on the Vie Scope in patients with expected easy airways is limited, and it remains unclear whether it can be used effectively for routine airway management by non-otorhinolaryngologist physicians [6]. Hence, we assumed that the first-attempt intubation success rate, intubation time, overall intubation success rate, and glottis visualization, according to Cormack-Lehane classification, with the Macintosh are superior to the Vie Scope in patients with expected easy airways, regardless of the use of personal protective equipment (PPE) and the underlying cause that necessitates airway management.

## Materials and methods

Ethical considerations

The study protocol was approved by the Scientific Committee of the General Hospital of Patras (reference number: 141/09.07.2023) on July 26, 2023. The committee waived the necessity for written informed consent given that the strategies employed in both study groups are considered components of standard care and the study involved patients undergoing elective and non-elective surgery or being treated in the emergency setting. The study was registered on ClinicalTrials.gov (NCT06149338) on November 29, 2023, and was conducted in accordance with the principles of the Declaration of Helsinki and the guidelines of Good Clinical Practice.

Study design

A single-blind, randomized, prospective, superiority clinical trial was conducted from January 26, 2024, to August 21, 2024. The same anesthesiology resident performed all intubations after achieving at least 50 attempts with each of the examined laryngoscopes before patient enrollment. A similar learning curve was determined for both laryngoscopes to eliminate any differences in familiarization and competency with each laryngoscope. Moreover, the limited data for the Vie Scope demonstrated its short learning curve, highlighting the importance of previous practice with straight-blade devices [5,6,9]. All interventions were supervised by an experienced anesthesiologist consultant, who instructed the airway operator to use the appropriate PPE in cases of certified or possible COVID-19 infection.

The current study included both male and female patients with a similar ratio. In addition to gender, age greater than 18 years old and body mass index (BMI) between 18.5 and 30 kg/m² were considered as inclusion criteria. A positive or negative rapid test for COVID-19, as well as possible patient contamination, was also documented without affecting patient eligibility. All patients underwent an adequate airway assessment, including a comprehensive airway history and physical examination, to identify those with an anticipated easy airway [2,10]. The chin protrusion (upper lip bite test), thyromental distance, and Mallampati score were primarily evaluated due to their efficacy in detecting an expected difficult airway [2]. Therefore, the higher the patient could bite the upper lip with the lower incisors, the higher the thyromental distance above three ordinary fingerbreadths, and Mallampati scores of 1 or 2 were positive predictive factors of an expected easy airway [2,10,11]. Despite the high sensitivity of the upper lip bite test, the low sensitivity of other tests, such as the Mallampati score, confined airway assessment in emergency settings, and inadequate patient compliance, necessitated the inclusion of the Cormack-Lehane classification as a recruitment criterion of this study [10,11]. According to this classification, grades 1, 2a, and 2b after direct laryngoscopy were compatible with easy endotracheal intubation, thereby waiving patient eligibility in the present study [12]. Consequently, patients aged under 18 years old with a BMI less than 18.5 kg/m² or more than 30 kg/m², an inability to place mandibular incisors anterior to maxillary ones, a thyromental distance below three ordinary fingerbreadths, Mallampati scores of 3 or 4, and Cormack-Lehane classification grades of 3 or 4 were excluded from this clinical trial.

Randomization of the patients was initiated before anesthesia induction and after the anesthesiology trainee was assigned to each patient, either in the operating room or in the emergency setting. Sealed, opaque envelopes containing pseudonymized demographic data and the medical background of each participant were used to allocate participants 1:1 for either the Vie Scope or Macintosh laryngoscope. The patients remained blinded to the intervention procedure, which was not feasible for the airway operator because of the study's design.

**Figure 1:**
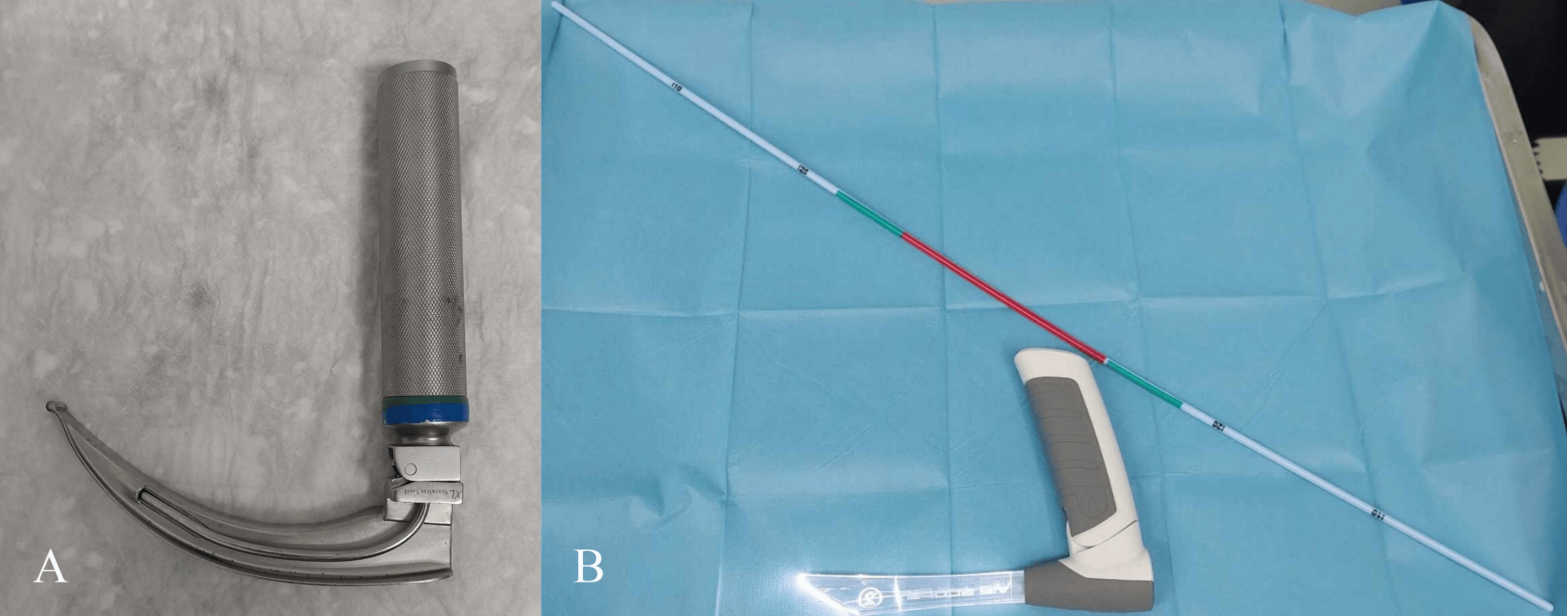
Macintosh laryngoscope (A) and Vie Scope laryngoscope with the VOIR Bougie (B)

Patients randomized to the control group underwent direct laryngoscopy and subsequent intubation with the Macintosh (Figure 1). The size of the blade (3 or 4) was determined at the discretion of the anesthesiologist trainee. The airway operator implemented a midline approach for tongue displacement from the area of view and placed the tip of the Macintosh blade in the epiglottic vallecula to achieve indirect elevation of the epiglottis and exposure of the vocal cords, followed by endotracheal intubation [4,13].

On the other hand, patients randomized into the intervention group received direct laryngoscopy and endotracheal intubation using the Vie Scope (Figure 1). The introduction of the Vie Scope into the patient's mouth can be feasible by either of the mouth corners, depending on the patient's anatomical characteristics [14,15]. A gentle back tilt of the head was followed by the application of the paraglossal or retromolar approach to align the oral, pharyngeal, and laryngeal axes [6,14]. The Vie Scope was forwarded until the glottis was visible by directly elevating the epiglottis. A 15-Fr bougie with colorful bands, the VOIR Bougie was inserted endotracheally through the cylindrical intubation channel of the Vie Scope. Subsequently, the Vie Scope was carefully removed, followed by the placement of an endotracheal tube (ETT) over the bougie [15,16]. Eventually, the bougie was also extracted [15,16].

The ETT cuff was inflated until no air leak from the trachea was detected, while the patients' ventilation verified correct ETT placement in both groups. The airway operator selected the size and type of the ETT. Additionally, the laryngeal view was evaluated under direct laryngoscopy using each laryngoscope according to the Cormack-Lehane grading system. The airway operator was prompted to perform external laryngeal manipulation, such as the backward, upward, and rightward pressure (BURP) technique, or use assistant equipment, including bougies, stylets, forceps, lubricant gel, or spray, to optimize the laryngeal view and facilitate successful intubation. However, the supervisor anesthesiology consultant was involved either after two failed intubation attempts or whenever the situation required the intervention of an experienced anesthesiologist.

Outcome measures

The success rate of the first intubation attempt and the required mean intubation time were considered the primary outcome measures. The intubation time was defined as the time interval from the insertion of the laryngoscope into the patient's mouth until the inflation of the ETT cuff in each patient. In contrast, the overall intubation success rate and glottis visualization, as determined by the Cormack-Lehane classification, were secondary outcomes.

Statistical analysis

The statistical analysis was performed using SAS version 9.4 (SAS Institute, Cary, NC, USA) and Microsoft Excel 2007 (Microsoft Corp., Redmond, WA, USA) for data analysis and presentation. For descriptive statistics, quantitative variables were presented as means and standard deviations (SD), as well as medians and interquartile ranges (IQR). Qualitative variables were calculated using relative frequencies and relevant percentages. Furthermore, the Kruskal-Wallis test was applied for the inferential part of the analysis, specifically for analyzing numerical data, as they did not follow a normal distribution, according to the Kolmogorov-Smirnov test. The x² test and, if required, Fisher's exact test were performed for categorical data. All tests were two-sided, and confidence intervals (CIs) were determined at a 95% confidence level; thus, the significance cut-off level was set at p<0.05.

## Results

**Figure 2:**
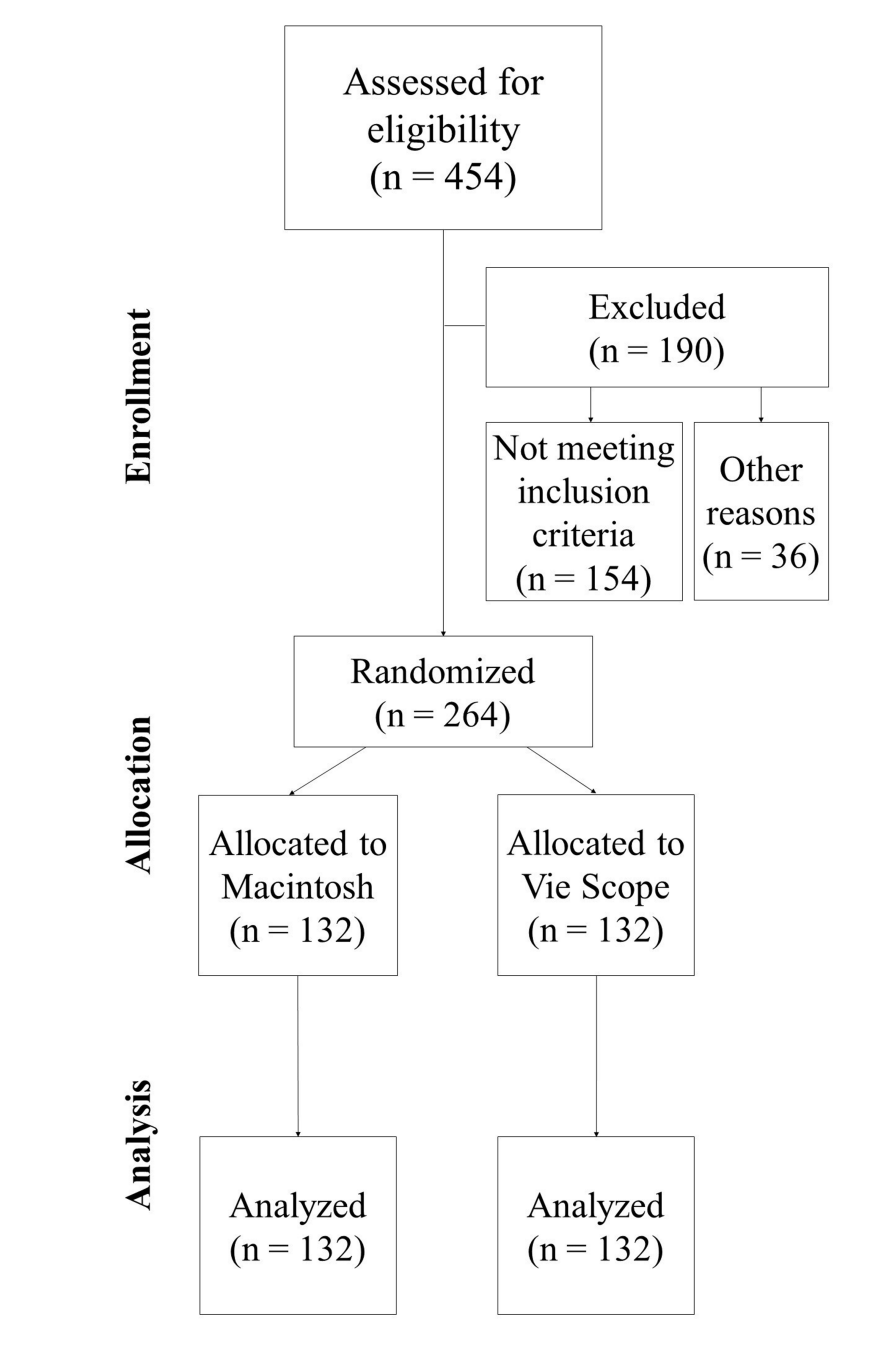
CONSORT study flowchart CONSORT: Consolidated Standards of Reporting Trials

**Figure 3:**
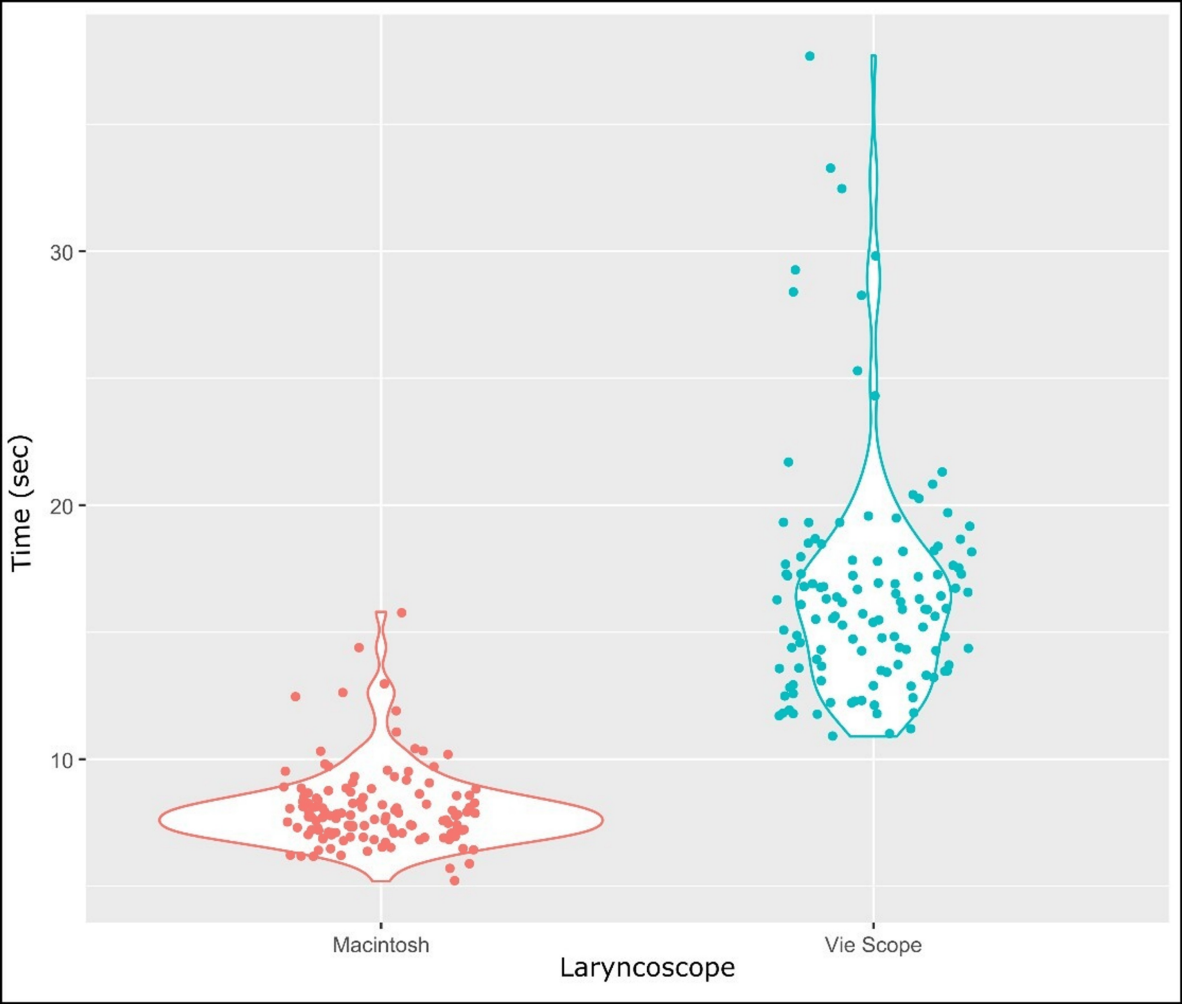
Violin plot of the required mean intubation time in the Vie Scope and Macintosh laryngoscope groups

From January 26, 2024, to August 21, 2024, 264 patients were randomly assigned to receive intubation either via the Macintosh laryngoscope or the Vie Scope. The grading of the glottis view, according to the Cormack-Lehane classification, was performed by the same trainee airway operator, who also confirmed successful intubation in all cases. An overview of patient inclusion is illustrated in Figure 2, while descriptive data about the enrolled patients are summarized in Tables 1-2. No statistically significant differences were noted regarding the features of the two laryngoscope groups (Table 3). The required intubation time was shorter with the Macintosh than with the Vie Scope (median (IQR): 7.80 (1.50) and mean (SD): 8.08 (5.34) seconds vs. median (IQR): 15.65 (3.90) and mean (SD): 16.65 (5.02) seconds, differences in means: 8.60 seconds, 95% CI: -∞ to 7.9, p<0.0001), as shown in the violin plot (Figure 3), complying with the superiority criteria of the Macintosh over the Vie Scope. The use of the Macintosh had a first-attempt intubation success rate of 100% (132/132) compared with 90.15% (119/132) for the Vie Scope, with an estimated p-value of 0.0002 and a relative risk (RR) of 0.90 (95% CI: 0.85-0.95). In the Vie Scope group, 13 patients were intubated on the second attempt after a failed first attempt; five of them used the Vie Scope, and the remaining eight patients used the Macintosh. Regarding the overall intubation success rate, the Macintosh showed a higher rate (132/132, 100%), regardless of the number of attempts, compared with the Vie Scope (124/132, 93.94%) with p=0.0041 and RR: 0.94 (= 1/1.06, 95% CI: 0.90-0.98). As the RR was lower than 1, the superiority of the Macintosh over the Vie Scope for the parameters mentioned above was justified. Lastly, no statistically significant difference was detected in the reported degree of glottis visualization between the two laryngoscope groups, as determined by the Cormack-Lehane classification (p=0.7895). An overview of the statistical data is presented in Table 4.

**Table 1:** Descriptive data of the enrolled patients regarding age, BMI, and intubation time BMI: body mass index, IQR: interquartile range, SD: standard deviation

Features	Values		
	Mean (SD)	Median (IQR)	Minimum, maximum
Age	61.98 (16.94)	65 (24.5)	18, 93
BMI (kg/m^2^)	24.5 (2.11)	24.4 (2.85)	19.6, 30
Intubation time (sec)	12.23 (5.46)	11.15 (8.1)	5.2, 37.7

**Table 2:** Descriptive data of the enrolled patients in the Vie Scope and Macintosh laryngoscope groups

Features	Values
Number of cases	
Macintosh	132
Vie Scope	132
Gender	
Male	134 (50.76%)
Female	130 (49.24%)
Mallampati score (%)	
1	137 (51.89%)
2	82 (31.06%)
Infeasible evaluation	45 (17.04%)
Cormack-Lehane classification (%)	
1	202 (76.52%)
2a	41 (15.53%)
2b	21 (7.95%)
Intubation conditions (number of cases (%))	
Urgent	106 (40.15%)
Elective	158 (59.85%)
Number of successful intubations (%)	264/264 (100%)
Intubation attempts (number of cases (%))	
One	251 cases (95.08%)
Two	13 cases (4.92%) from the Vie Scope group, five of them intubated via the Vie Scope, and eight of them via the Macintosh
Encountered intubation difficulties (number of cases (%))	
Yes	35 (13.26%)
No	229 (86.74%)

**Table 3:** Comparison of the features of the Vie Scope and Macintosh laryngoscope groups IQR: interquartile range, BMI: body mass index

Feature	Macintosh group (n=132)	Vie Scope group (n=132)	p
Age (median (IQR))	63.5 (26.5)	67 (21.5)	0.1990
BMI (kg/m^2^) (median (IQR))	24.2 (2.9)	24.65 (2.55)	0.9065
Gender - male (%)	64 (48.48%)	70 (53.03%)	0.4601
Number of cases with urgent airway management (%)	47 (35.61%)	59 (44.70%)	0.1319
Number of cases with encountered intubation difficulties (%) - number of urgent cases	15 (11.36%) - 9/15	20 (15.15%) - 8/20	0.3642

**Table 4:** Summary of the results after the utilization of the Vie Scope and Macintosh laryngoscope CI: confidence interval, IQR: interquartile range, NA: not applicable, RR: relative risk, SD: standard deviation

Feature	Macintosh group (n= 132)	Vie Scope group (n= 132)	p	RR (95%CI)
Overall successful intubation rate with the same device, regardless of the number (%)	132 (100)	124 (93.94)	0.0041	1.06 (1.02-1.11)
First-attempt intubation success rate (%)	132 (100)	119 (90.15)	0.0002	1.1 (1.05-1.17)
Intubation time - means (SD) (sec)	8.08 (5.34)	16,65 (5.02)	<0.0001	ΝΑ
Intubation time - median (IQR) (sec)	7.80 (1.50)	15.65 (3.90)	<0.0001	NA
Cormack-Lehane classification (%)	-	-	0.7895	NA
1	100 (75.76)	102 (77.27)	-	-
2a	20 (15.15)	21 (15.91)	-	-
2b	12 (9.09)	9 (6.82)	-	-
Mallampati score (%)	-	-	0.1549	RR: 1.49 (0.85-2.59)
1	82 (66.67)	55 (57.29)	-	-
2	41 (33.33)	41 (42.71)	-	-

## Discussion

This clinical study aimed to present comparative data on the expected ease of airway management using the Macintosh laryngoscope and the Vie Scope. Achieving endotracheal intubation with the Macintosh was 8.60 seconds faster than using the Vie Scope. It was correlated with a higher first-attempt intubation success rate (100% vs. 90.15%, respectively), indicating the superiority of the Macintosh over the Vie Scope for primary outcomes.

The delayed intubation process via the Vie Scope was attributed to the mandatory use of the bougie, over which the ETT was forwarded [5,7]. The majority of the published studies estimated mean intubation times of approximately 30 and over 35 seconds for the Vie Scope and the Macintosh, respectively, except for a simulation trial with required mean intubation times of 20.8 (8.1) and 36.3 (10.1) seconds for the Macintosh and the Vie Scope, respectively [7,15-17]. Wieczorek et al. reported equivalent results (29.8 (3.6) vs. 33.9 (5.4) seconds for the Vie Scope and the Macintosh, respectively) during pediatric airway management while using PPE. In contrast, no statistically significant difference was noted in the absence of PPE use between the compared laryngoscopes [18]. In contrast, our clinical study included nine COVID-19-infected patients, five of whom were successfully intubated on the first attempt with the Macintosh, and the other four with the VIE Scope, with the required times ranging between 7.7 and 15.8 seconds and 15.9 and 28.3 seconds, respectively. Furthermore, the implementation of the Vie Scope was associated with higher first-attempt intubation success rates compared with the Macintosh, exceeding 91%, as reported in the literature [5,15,16,18]. However, Ratajczyk et al. demonstrated lower first-attempt intubation success rates in their study (65% for the Vie Scope vs. 60% for the Macintosh). In contrast, similar percentages for the Macintosh and the Vie Scope were documented during airway management in simulators by anesthesiology specialists (91% vs. 89%, respectively) [7,17].

Nonetheless, previously mentioned studies mainly focused on difficult airway management, and intubation attempts were performed either by paramedics, individuals without clinical experience mainly wearing PPE, or on simulators [5,7,15-18]. In the present study, the trainee's pre-existing training and experience facilitated the successful management of all patients with an expected easy airway, despite sparse data on the inpatient use of the Vie Scope [5,6]. In addition, the anatomical and physiological differences between simulators and real patients, as well as the inability of manikin studies to incorporate critical factors such as stress and cognitive overload, often result in outcomes that are challenging to interpret or even inconsistent with those from human studies [7,19].

Moreover, the reported predominance of the Macintosh over the Vie Scope regarding the overall intubation success rate (132/132, 100% vs. 124/132, 93.94%, respectively), contradicting the existing literature, can be equally explained [15,16]. Difficulties in securing an airway were encountered in both the control and intervention groups (15 cases (11.36%) vs. 20 cases (15.15%), respectively). The presence and aspiration of blood or secretions in the upper airway, especially the inspissated ones; cervical spine fractures that prevent the alignment of the pharyngeal and the laryngeal axis; and teeth protrusion, associated with dental trauma if direct pressure is applied on them by the laryngoscope, were the main difficulties faced by the airway operator when using the Macintosh and the Vie Scope [20-22]. BURP manipulation enabled endotracheal intubation in most cases, despite equivocal data regarding its efficacy. In contrast, endotracheal advancement of the bougie under direct vision resulted in successful intubation without complications [6,23-26].

Nevertheless, the insufficient application of the paraglossal technique when introducing the Vie Scope into the patient’s mouth resulted in either failure or delay of the first intubation attempt. Given the trainee’s competency level and aim to avoid dental injury while using the teeth as a fulcrum during laryngoscopy, the presence of complete dentures and lack of available tooth guard most likely contributed to this finding [20]. Furthermore, the restricted maneuvers into the upper airway, along with the one-sized straight blade of the Vie Scope, compared with the size variety of the Miller blade, led to difficult or infeasible epiglottis elevation [21]. In addition, the inability to railroad the ETT over the bougie, irrespective of the ETT size and the inflated cuff, can be attributed to the ETT’s impingement on the arytenoid cartilage, which was overcome in some cases by 180° rotation of the ETT and spraying lidocaine 2% as a lubricant [6,8]. However, this was not confirmed by repetitive laryngoscopy using the Vie Scope. As a result, 13 patients in the Vie Scope group were successfully intubated after the second attempt, with five of them using the Vie Scope. In contrast, the other eight patients deteriorated clinically during intubation, and the trainee was instructed to use the Macintosh, as he was more competent with it [27].

Additionally, 59/132 patients in the Vie Scope group and 47/132 in the Macintosh group received urgent endotracheal intubation, with the airway operator facing difficulties in fewer patients in the Vie Scope group (8/20) compared with the Macintosh group (9/15). Grade 1 visualization of the vocal cords was also achieved in more patients in the Vie Scope group (46/59) than in the Macintosh group (29/47) in emergency settings. The focused transmission of intense light onto target anatomical structures, combined with its sealed tube and angled end, enables its successful utilization in both urgent inpatient and outpatient settings while ensuring an optimal laryngeal view unaffected by tissue prolapse and upper airway secretions [5,6,15]. Therefore, our study showed a similar degree of visualization of the glottis between the two laryngoscopes, as grades 1 and 2a were achieved in approximately 75% and 15% of the sample groups, respectively. Grade 2b visualization of the glottis was noted in less than 10% of each sample group, despite its direct elevation by straight blades being correlated with better visualization of the vocal cords during difficult airway management compared to Macintosh and videolaryngoscope curved blades [6-8,28-30]. These findings support the inclusion of the Vie Scope as an effective alternative method for airway management.

The primary constraint of this study was the limited experience of the primary airway operator, who was a trainee anesthesiologist. Although the anesthesiologist trainee had completed a minimum of 50 successful intubations with each laryngoscope before the study, he was more comfortable and experienced with the Macintosh, as it is typically the first laryngoscope with which anesthesiologist residents become familiar.This familiarity may have influenced the different intubation times observed among the laryngoscopes used in the study. Therefore, the outcomes were not indicative of the use of the Macintosh and the Vie Scope by experienced and well-trained anesthesiologists during the establishment of easy airways. Concerning glottis visualization, patients with Cormack-Lehane grades 3 and 4 were excluded from the study after the completion of the intubation process, which creates a risk of post-randomization bias. Moreover, patients in both groups were not reassessed after endotracheal intubation by direct laryngoscopy to detect any potential complications, such as trauma of the oral and pharyngeal mucosa and the laryngeal structures. Moreover, the current study was limited to the management of easy airways among adults. Hence, safe conclusions could not be drawn regarding the efficacy and the laryngeal view provided by both laryngoscopes for difficult airway settings in both adult and pediatric patients.

## Conclusions

This clinical trial demonstrated that the Macintosh laryngoscope is superior to the Vie Scope in terms of first-attempt and overall endotracheal intubation success rates, as well as the mean time required for intubation during the establishment of an anticipated easy airway. Nevertheless, no statistically significant intergroup differences were noted regarding the degree of glottis visualization between the two laryngoscopes, thus reinforcing the inclusion of the Vie Scope as a feasible tool for securing an airway. However, more clinical studies are warranted to provide important information about its safe and effective use in both adult and pediatric patients, as well as appropriate learning curves for both laryngoscopes.
